# Inactivation of SAG E3 Ubiquitin Ligase Blocks Embryonic Stem Cell Differentiation and Sensitizes Leukemia Cells to Retinoid Acid

**DOI:** 10.1371/journal.pone.0027726

**Published:** 2011-11-15

**Authors:** Mingjia Tan, Yun Li, Ruiguo Yang, Ning Xi, Yi Sun

**Affiliations:** 1 Division of Radiation and Cancer Biology, Department of Radiation Oncology, University of Michigan, Ann Arbor, Michigan, United States of America; 2 Key Laboratory of Marine Drugs, Chinese Ministry of Education, and School of Medicine and Pharmacy, Ocean University of China, Qingdao, China; 3 Department of Electrical and Computer Engineering, Michigan State University, East Lansing, Michigan, United States of America; Penn State Hershey Cancer Institute, United States of America

## Abstract

Sensitive to Apoptosis Gene (SAG), also known as RBX2 (RING box protein-2), is the RING component of SCF (SKP1, Cullin, and F-box protein) E3 ubiquitin ligase. Our previous studies have demonstrated that SAG is an anti-apoptotic protein and an attractive anti-cancer target. We also found recently that *Sag* knockout sensitized mouse embryonic stem cells (mES) to radiation and blocked mES cells to undergo endothelial differentiation. Here, we reported that compared to wild-type mES cells, the *Sag^−/−^* mES cells were much more sensitive to all-trans retinoic acid (RA)-induced suppression of cell proliferation and survival. While wild-type mES cells underwent differentiation upon exposure to RA, *Sag^−/−^* mES cells were induced to death via apoptosis instead. The cell fate change, reflected by cellular stiffness, can be detected as early as 12 hrs post RA exposure by AFM (Atomic Force Microscopy). We then extended this novel finding to RA differentiation therapy of leukemia, in which the resistance often develops, by testing our hypothesis that SAG inhibition would sensitize leukemia to RA. Indeed, we found a direct correlation between SAG overexpression and RA resistance in multiple leukemia lines. By using MLN4924, a small molecule inhibitor of NEDD8-Activating Enzyme (NAE), that inactivates SAG-SCF E3 ligase by blocking cullin neddylation, we were able to sensitize two otherwise resistant leukemia cell lines, HL-60 and KG-1 to RA. Mechanistically, RA sensitization by MLN4924 was mediated via enhanced apoptosis, likely through accumulation of pro-apoptotic proteins NOXA and c-JUN, two well-known substrates of SAG-SCF E3 ligase. Taken together, our study provides the proof-of-concept evidence for effective treatment of leukemia patients by RA-MLN4924 combination.

## Introduction

SAG, also known as RBX2, ROC2 (Regulator of Cullins) or RNF7 (RING finger protein-7), was originally cloned in our laboratory as a redox-inducible antioxidant protein [1], and later characterized as the second RING family member of the SCF E3 ubiquitin ligase (for review, see [2]). We and others have previously shown that in cell culture systems, SAG overexpression inhibited apoptosis induced by various agents, including redox [1], [3], nitric oxide [4], ischemia/hypoxia [5], heat shock [6], neurotoxins and 1-methyl-4-phenylpyridinium [7], and UV-irradiation [8]. SAG over-expression also promoted the S-phase entry and cell growth under serum starved conditions [9]. Furthermore, SAG transgenic expression in mouse skin inhibited tumor formation at the early stage, but enhances tumor growth at the later stage in a DMBA-TPA carcinogenesis model [10]. On the other hand, SAG knockdown by anti-sense or siRNA oligoes inhibited tumor cell growth [11], and enhanced apoptosis induced by etoposide and TRAIL [12]. The *Sag* knockout in mouse caused embryonic lethality, which is associated with growth retardation and other developmental defects [13], whereas *Sag* knockout in mES cells induced radiosensitization [14], and blocked their endothelial differentiation [13]. These cellular functions were mediated via its antioxidant activity by scavenging ROS [1], [4], [6], and via its E3 ubiquitin ligase activity by promoting the degradation of p27, c-Jun, pro-caspase-3, IκBα, HIF-1α, and NOXA in a cell context dependent manner [9], [10], [12], [15], [16], [17]. Importantly, SAG was overexpressed in carcinomas of lung, colon, stomach and liver, which was associated with poor prognosis in lung cancer patients [11], [17], [18]. The findings suggest that SAG may play a role in human tumorigenesis and could serve as an anticancer target.

Retinoic acids (RAs) are natural and synthetic derivatives of vitamin A [19]. The binding of RAs to their nuclear receptors, including RAR or RXR, PPAR in the homo- and hetero-dimer formats [20], caused transactivation of more than 500 genes [21] that regulates many signaling pathways [22], and cellular processes including embryonic development, organogenesis, cell homeostasis, cell growth, differentiation and apoptosis [23], [24], [25], [26]. Given its activity in inducing differentiation, apoptosis, and growth arrest, RA has been long used for the treatment and prevention of various types of human cancers [19], [27], [28], [29], [30], particularly as a differentiation therapy agent for the treatment of AML (Acute Myeloid Leukemia) [31], [32]. Significantly, RA is very effective for the treatment of one type AML, acute promyelocytic leukemia (APL) with a cure rate in more than 75% of patients [26]. However, the complete success of this therapy was hindered by the development of RA resistance with subsequent disease relapses [30], demanding combinational therapies, most effectively with arsenic trioxide [33], and other chemotherapeutic drugs, as well as targeted therapies, including TRAIL [26], [27], [30].

Here we report that cellular sensitivity to RA is significantly increased upon inactivation of SAG E3 ubiquitin ligase via genetic deletion or pharmacological inhibition. Specifically, instead of inducing differentiation in wild type mES cells, RA induced apoptosis in *Sag^−/−^* mES cells. In two SAG high-expressing AML lines, HL60 and KG-1, MLN4924, a small molecule inhibitor of SAG E3, effectively sensitized otherwise resistant cells to RA via induction of apoptosis which is associated with accumulation of pro-apoptotic proteins, c-JUN and NOXA. Thus, our study reveals that SAG is a RA sensitizing target and provides the first proof-of-concept evidence that MLN4924 can be further developed as a RA chemo-sensitizer for the treatment of AML.

## Results

### Sag deletion sensitized mouse embryonic stem cell to RA

**Figure 1:**
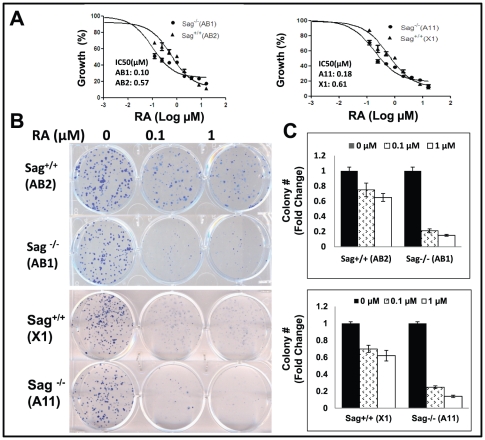
Sag deletion sensitizes mES cells to all-trans Retinoid Acid (RA). Two independent pairs of mES cells with genotypes of Sag^+/+^ and Sag^−/−^ were seeded in 96-well plate in triplicate and treated with various concentrations of RA for 24 hrs. Cell viability was measured by ATP-lite assay. The IC50 curve was generated and IC50 value calculated by the Prism software (**A**). Cells were seeded in 6-well plate in duplicate and treated with DMSO vehicle control or RA (0.1 and 1 µM) for 6 days. The plates were stained (**B**) and the colonies with more than 50 cells were counted and plotted (**C**). Shown is x ± SEM from three independent experiments.

Our recent study showed that Sag^−/−^ mES cells failed to undergo endothelial differentiation to form cystic embryoid bodies upon the withdrawal of LIF (leukemia inhibitory factor) from culture media [13]. Here we determined if Sag^−/−^ mES cells were also defective in RA-induced differentiation. Using two independent pairs of mES clones with Sag^+/+^ vs. Sag^−/−^ background [14], we first determined the effect of Sag deletion on cell proliferation and survival in the absence and presence of RA. While Sag deletion had no effect on monolayer and clonogenic growth, it significantly sensitized mES cells to RA. The IC50 value (the concentration that caused 50% growth inhibition), determined by ATPlite-based cell proliferation assay, was 3.4- to 5.7-fold lower in Sag^−/−^ mES cells than Sag^+/+^ mES cells (Fig. 1A). Likewise, the clonogenic survival assay showed a similar 3- to 4-fold sensitization to RA upon Sag deletion (Fig. 1B, 1C). Thus, Sag deletion sensitizes mES cells to RA.

### Sag deletion impaired ES cell differentiation induced by RA

**Figure 2:**
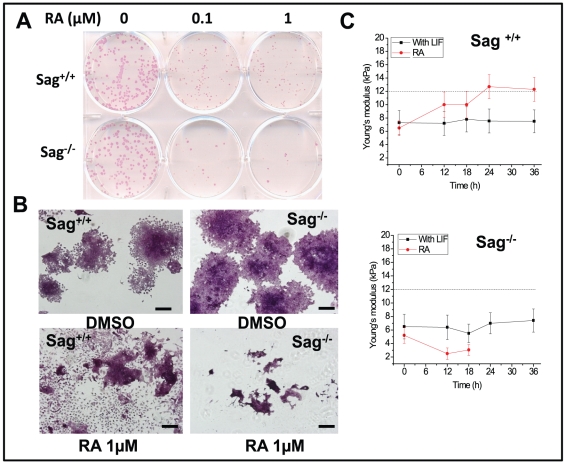
Sag deletion blocks RA-induced differentiation. Sag^+/+^ and Sag^−/−^ mES cells were seeded in 6-well plate and treated with RA at indicated concentrations for 6 days. Colonies were stained with AP (**A**) and counter-stained with hematoxylin (**B**), followed by photography. Bar size = 50 nm. Cells were seeded in geletin-coated glass coverslips and cellular stiffness was measured by AFM nanomechanical analysis at 0, 12, 18, 24 and 36 hrs post RA (1 µM) treatment. Shown is x ± SD from three independent experiments (**C**).

We next used one representative pair of mES cells (AB1/Sag^−/−^vs. AB2/Sag^+/+^) to determine if Sag^−/−^ mES cells failed to undergo RA-induced differentiation. We plated mES cells in low density in gelatin-coated plate, treated with RA at 0, 0.1 and 1 µM for 6 days, and then stained with alkaline phosphatase (AP), a marker for undifferentiated ES cells [34]. In the absence of RA treatment, Sag^−/−^ and Sag^+/+^ mES cells showed no difference in colonic growth and in the maintenance of the undifferentiated status (Fig. 2A). In the presence of RA treatment (at both 0.1 and 1.0 µM), however, the proliferation of Sag^−/−^ mES cells were severely inhibited with remarkable reduction in the number of undifferentiated colonies (Fig. 2A). Examination microscopically revealed many differentiated cells from Sag^+/+^ mES cells, whereas only scarce cell debris were observed in Sag^−/−^ mES cells (Fig. 2B), suggesting a robust cell killing. We also used AFM (Atomic Force Microscopy), which measures the cell mechanics, to determine potential early changes during RA-induced differentiation. As shown in Figure 2C, the cellular stiffness was similar between Sag^+/+^ and Sag^−/−^ mES cells at about 5–7 kPa and was remained constant for 36 hrs in the presence of LIF (to maintain undifferentiated status). Upon addition of RA to induce differentiation, the cellular stiffness increased in Sag^+/+^ mES cells, starting at 12 hrs and reaching the peak of 12 kPa at 24 hr. In contrast, the cellular stiffness in Sag^−/−^ mES cells decreased by 50% to 2.5 kPa at 12 hrs and fell beyond detection level after 18 hrs, likely due to the initiation of cell death process. The result clearly demonstrated that physical changes, as reflected by cellular stiffness, occur at the very early stage during RA-induced differentiation, long before any morphological changes which take days to occur.

### Sag deletion enhanced RA induced apoptosis

**Figure 3:**
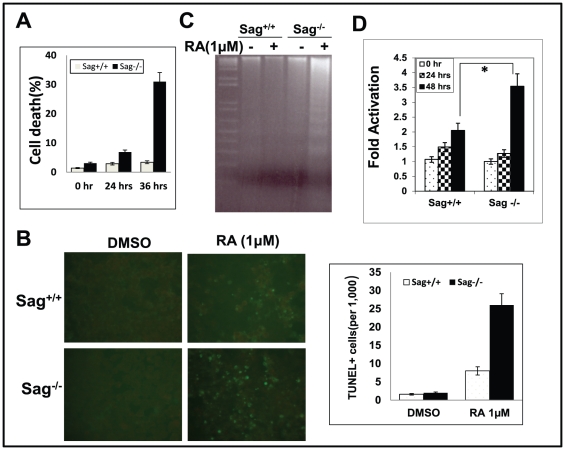
Sag deletion sensitizes mES cells to RA-induced apoptosis. mES cells with genotype of Sag^+/+^ and Sag ^−/−^ were treated with DMSO control or 1 µM RA for indicated time periods, followed by trypan blue staining (**A**), TUNEL staining at 36 hrs (**B**, left panel), with quantification of TUNEL positive cells graphed (**B**, right panel), DNA fragmentation assay at 36 hrs (**C**), and caspase-3 activity assay at the indicated time point (**D**). *, *p*<0.05.

We next used several independent assays to determine the nature of RA-induced cell death in Sag^−/−^ mES cells. Both trypan blue exclusion assay and TUNEL showed that about 30% of Sag^−/−^ mES cells were dead or stained TUNEL positively after 36 hrs of RA treatment, compared to ∼3% of Sag^+/+^ mES cells (Fig. 3A, 3B). DNA fragmentation assay, a hallmark of apoptosis, showed a remarkable induction of DNA ladders in Sag^−/−^, but not in Sag^+/+^ mES cells (Fig. 3C). Likewise, caspase-3 activity assay also showed a significantly higher enzymatic activation in Sag^−/−^ than in Sag^+/+^ mES cells (Fig. 3D). Taken together, our data suggest that Sag deletion triggers mES cells to undergo apoptosis, instead of differentiation in response to RA.

### Sensitization of AML cells to RA by MLN4924, a SAG-SCF E3 ligase inhibitor

**Figure 4:**
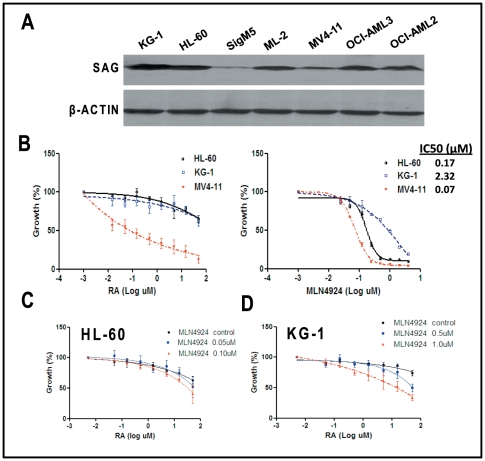
Correlation between SAG overexpression and RA resistance in AML cell lines and their sensitivity to RA and/or MLN4924. Proteins were extracted from 7 AML lines and subjected to immunoblotting using antibody against SAG with β-actin as the loading control (**A**). Cells were seeded in 96-well plate in triplicate and treated with various concentrations of RA (**B**, left panel), MLN4924 (**B**, right panel) or in combination at indicated concentrations of each drug for 48 hrs (**C**, HL-60 cells) and (**D**, KG-1 cells), followed by ATP-lite cell viability assay. Values were normalized to the untreated control. Shown is x ± SEM from three independent experiments with IC50 curve generated and IC50 value calculated using the Prism software.

RA was used as a differentiation therapy for AML patients and the disease relapsed upon development of RA resistance [30], [31]. We therefore tested our working hypothesis that inactivation of SAG E3 ligase may sensitize AML cells to RA. We first determined SAG protein levels in seven AML lines and found SAG expression varied among these lines (Fig. 4A). We then determined a potential correlation between SAG levels and RA sensitivity using two SAG high-expressing lines, HL-60 and KG-1 and one SAG low-expressing line, MV4-11 (Fig. 4A), and found a good correlation with SAG high-expressing cells being much more resistant to RA (IC50>50 µM in HL-60 and KG-1 cells vs. IC50<0.1 µM in MV4-11 cells) (Fig. 4B, left panel). We then used MLN4924, a small molecule inhibitor of NEDD8-Activating Enzyme (NAE), that inactivates SAG-SCF E3 ligase by blocking cullin neddylation [35] for potential RA sensitization. Before the combination study, we determined the cytotoxicity of MLN4924 as a single agent against three AML lines and found again, SAG high-expressing lines were more resistant to MLN4924 with IC50 of 0.17 µM (HL-60), 2.32 µM (KG-1) and 0.07 µM (MV4-11), respectively (Fig. 4B, right panel). In RA sensitization experiment, we used two concentrations of MLN4924 that caused ∼20–30% cytotoxicity when used alone (0.05–0.1 µM for HL-60, and 0.5–1 µM for KG-1 cells) in combination with various concentrations of RA, ranging from 5 nM to 50 µM. We observed a dose-dependent additive or synergetic effect on growth suppression of HL-60 cells (Fig. 4C) or KG-1 cells (Fig. 4D). Thus, SAG inhibitor MLN4924 could sensitize otherwise resistant AML cells to RA.

### RA-MLN4924 combination enhanced apoptotic cell death

**Figure 5:**
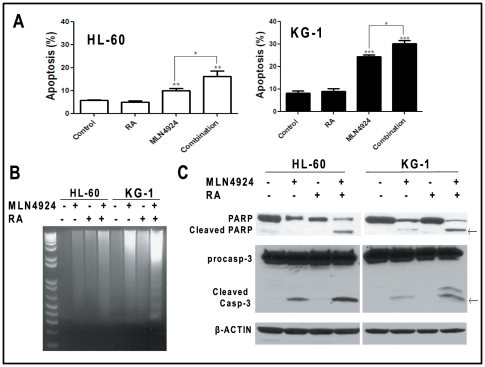
MLN4924 induces RA sensitization by inducing apoptosis. Cells were treated with DMSO control, RA (1.5 µM), MLN4924 (0.1 µM for HL-60, 1.0 µM for KG-1) alone or in combination for 24 hrs (HL-60) or 36 hrs (KG-1), followed by FACS analysis for apoptotic sub-G1 population. *, *p*<0.05; **, *p*<0.01; ***, *p*<0.0001, compared to control (**A**). Cells were treated with indicated drugs at concentrations described above for 24 hrs, followed by DNA fragmentation assay (**B**) or immunoblotting using indicated antibodies (**C**).

Since Sag deletion sensitized mES cells to RA via apoptosis (Fig. 3), we reasoned that apoptosis would likely be the underlying mechanism for RA sensitization by MLN4924. Indeed, the sub-G1 apoptotic population by FACS analysis was significantly increased by the single treatment of MLN4924, but not RA. MLN4924-RA combination treatment further enhanced apoptosis in both lines (Fig. 5A). Likewise, DNA fragmented ladders (Fig. 5B) as well as cleavages of PARP and caspase-3 (Fig. 3C), the hallmarks of apoptosis, were more evidently seen in the combination group than in MLN4924 single treatment group, although RA alone failed to induce apoptosis. Thus, both HL-60 and KG-1 cells are resistant to RA, and MLN4924 confers RA sensitization by inducing apoptosis.

### RA-MLN4924 combination enhanced accumulation of pro-apoptotic proteins c-JUN and NOXA

**Figure 6:**
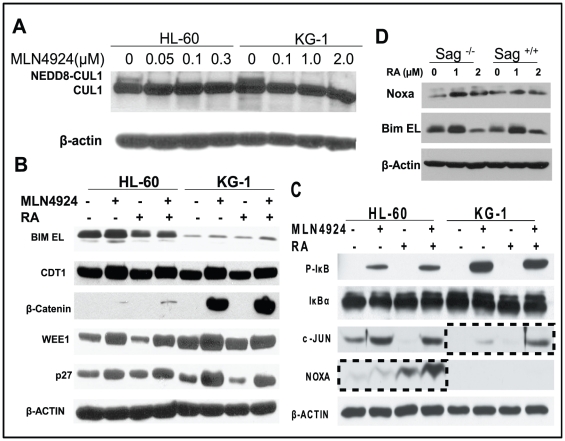
Induction of SAG-SCF E3 ligase substrates by RA and/or MLN4924. Cells were treated with indicated concentrations of MLN4924 for 24 hrs, followed by immunoblotting using cullin-1 antibody with β-actin as the loading control (**A**). Cells were treated with RA (1.5 µM), MLN4924 (0.1 µM for HL-60, 1.0 µM for KG-1) alone or in combination for 24 hrs, followed by immunoblotting using indicated antiobodies (**B&C**). mES cells were treated with RA at indicated concentrations for 24 hrs, followed by immunoblotting using NOXA antibody with β-actin as the loading control (**D**).

Since SAG is the RING component of SCF E3 ubiquitin ligase required for its ligase activity [36], we expected that SAG-SCF E3 inactivation by MLN4924 via cullin deneddylation would cause accumulation of SAG-SCF E3 ligase substrates. We further expected that MLN4924-RA combination would cause further increase in the levels of critical substrates, responsible for enhanced apoptosis. To this end, we first confirmed that MLN4924 treatment indeed caused NEDD8 removal from cullin-1 (Fig. 6A). We then determined potential accumulation of a subset of SAG-SCF E3 substrates known to regulate cell growth, cell cycle progression and apoptosis [37], [38], and found expected accumulation by MLN4924 of CDT1, β-catenin, WEE1, p27, phosphor-IκBα, c-JUN and NOXA, but not pro-apoptotic proteins BIM EL and IκBα (Fig 6B, 6C). Among all these substrates tested, only c-JUN in KG-1 cells and NOXA in HL-60 cells were further increased upon MLN4924-RA combination (Fig. 6D), indicating a correlation between further accumulations of c-JUN/NOXA and enhanced induction of apoptosis. In addition, we found that although c-Jun was not detectable in mES cells regardless of RA treatment (data not shown), Noxa, but not another pro-apoptotic protein Bim EL, known as SCF E3 substrate [39], did accumulate in Sag^−/−^, but not Sag^+/+^ mES cells upon RA treatment (Fig. 6D). These results further suggest an involvement of NOXA, a newly identified SAG substrate [17], in RA-induced apoptosis.

## Discussion

Our recent work showed that during mES cell differentiation induced by LIF withdrawal, Sag deletion had no effect on the formation of embryoid bodies, but prevented the endothelial differentiation to form blood island structure [13]. Here we determined the effect of Sag deletion on differentiation induced by RA, a powerful agent that triggers terminal differentiation of mES to many cell types [40]. We found that Sag deletion prevented mES cells from undergoing differentiation, rather triggered apoptosis instead. Although the molecular mechanism that governs the switch from RA-induced differentiation to apoptosis in the absence of Sag E3 ligase merits a future investigation, accumulation of its substrates, such as pro-apoptotic protein Noxa, is likely involved.

In this study, we also found for the first time that AFM, a valid tool in measurement of cell mechanics with incremental application in cell biology [41], can be used to monitor the process of RA-induced mES cell differentiation at very early stage. In wild type mES cells, we observed an increased cellular stiffness, starting at 12 hrs post RA treatment, long before any measurable change in cell morphology. In contrast, Sag^−/−^ mES cells showed a decrease in cellular stiffness, followed by its complete loss after 18 hrs, likely at the point when apoptosis is committed. This strike difference, observed at the very early stage of RA differentiation, suggests that the Sag-dependent “decision” of differentiation vs. apoptosis in response to RA, must occurs rapidly, likely involving gene transcription (RA effects) and rapid protein turn-over (Sag effects). The data also suggests that AFM measurement of cellular stiffness may be used as an early biomarker for cell differentiation.

Applying our finding of RA sensitization by Sag deletion in mES cells to AML, a human malignancy responsive to RA differentiation therapy, we observed a direct correlation of SAG overexpression and RA resistance. To mimic SAG deletion, we used MLN4924, a newly discovered small molecule inhibitor of NAE [35] that inactivates SAG-SCF E3 ligase by blocking cullin neddylation [35], [42], and found that combination of RA and MLN4924 caused significantly more apoptosis than each drug alone in two RA resistant AML cell lines. Mechanistically, we correlated enhanced apoptosis to higher level accumulation of c-JUN in KG-1 cells and of NOXA in HL-60 cells in drug combination. It is worth noting that MLN4924 was recently shown to be an effective apoptosis-inducing agent against AML lines, including MV4-11 and HL-60 [43].

It is well-established that c-JUN forms homo-dimer and hetero-dimer (with c-FOS) to compose active transcription factor AP-1 [44], and that AP-1 is involved in both induction and suppression of apoptosis in a cell context dependent manner [45]. It is also known that c-JUN could form a hetero-dimer with ATF-2 (Activating Transcription Factor-2), a stress responsive bZIP family of transcript factor with context-dependent proliferative as well as pro-apoptotic activities [46], [47]. Our observation that c-JUN is further accumulated in RA-MLN4924 treated cells with a consequent enhancement of apoptosis suggested its pro-apoptotic role. On the other hand, NOXA is a well-known BH3 domain-containing pro-apoptotic protein [48], which binds to anti-apoptotic proteins MCL1 and A1 to release their inhibitory binding with pro-apoptotic proteins BAX and BAK, thus inducing apoptosis [49]. Our recent study revealed that NOXA is a substrate of SAG E3 ligase that mediated apoptosis induced by SAG knockdown [17]. The fact that NOXA accumulates upon Sag deletion or inactivation in both mES and HL-60 cells in response to RA, strongly argue its apoptosis-causing role in these settings.

In summary, our study showed here that Sag E3 ligase determines the cell fate of mES cells to undergo differentiation or apoptosis in response to RA. We also validated that SAG, which is often overexpressed in human cancers [17], [50], is an attractive target for RA-mediated differentiation therapy in AML. Together with our previous work, demonstrating that SAG-SCF E3 ubiquitin ligase is an attractive anti-cancer and radiosensitizing target (for review, see [50], [51], [52], [53]), the small molecule inhibitor of SAG-SCF E3 ligase, such as MLN4924, which is currently under several phase I clinical trials [54], may have multiple applications in the treatment of human cancers.

## Materials and Methods

### Ethics Statement

No human participants. All animal procedures were approved by the University of Michigan Committee on Use and Care of Animals with an approval number 09701. Animal care was provided in accordance with the principles and procedures outlined in the National Research Council Guide for the Care and Use of Laboratory Animals.

### Generation and maintenance of Sag embryonic stem cells

Generation of ES cells from blastocysts were performed as described [55]. Briefly, blastocysts at the embryonic stage of E3.5 were isolated from *Sag^+/−^* females mated with *Sag^+/−^* males, and cultured on irradiated MEFs feeder cells in DMEM medium containing 15% fetal bovine serum, 0.1 mM β-mercaptoethanol, 50 IU/ml penicillin, 50 µg/ml streptomycin, 10^3^ u/ml ESGRO, and 12.5 µM PD98059. Inner cell mass outgrowths were trypsinized and cultured in 35-mm cell culture dishes in the same medium, but without antibiotics and PD98059. Embryonic stem cells were passaged on 0.1% gelatin-coated dishes to eliminate the feeder cells before genotyping and experimentation.

### Cells and cell culture

All AML cells (KG-1, HL-60, SigM5, ML-2, MV4-11, OCI-AML3 and OCI-AML2) were the gift from Dr. S. Malek at the University of Michigan. All the lines were originally purchased from ATCC and were cultured, according to ATCC suggested culture conditions.

### ATPlite growth assay and IC_50_ determination

Cells were seeded in 96-well plates in triplicates and treated with all trans-RA (Sigma) and/or MLN4924 (a gift from Millennium) at various concentrations for 24 hrs (mES) or 44 hrs (AML). Cell viability was measured with ATPlite kit (Perkin Elmer).

### Clonogenic assay

ES cells were seeded in six-well gelatin-coated plates in duplicate. Cells were treated next day with RA at various concentrations, followed by incubation at 37°C incubator for 5 to 7 days. Colonies were washed with PBS once and stained with brilliant blue G-250. The colonies with 50 or more cells were counted [14].

### Alkaline phosphatase (AP) staining

Sag^+/+^ and Sag^−/−^ mES cells were seeded in 6-well gelatin-coated plates, treated with DMSO vehicle control or RA at 0.1 µM or 1 µM for 6 days. The colonies were stained with Alkaline Phosphatase kits (Sigma-Aldrich) according to the manufacture' protocol. Briefly, after PBS wash, the colonies were fixed with fixative solution for 30 seconds, rinsed gently in water for 45 seconds, and then stained with alkaline-dye mixture by incubation at room temperature for 15 minutes. The colonies were rinsed, counterstained with hematoxylin, and evaluated microscopically.

### AFM (Atomic Force Microscopy) nanomechanical analysis

All the experiments for AFM measurement were performed in aqueous environment at room temperature on Bioscope (Bruker AXS, Santa Barbara, CA) using a Nanoscope IV controller. Once the mES cells seeded onto geletin-coated glass coverslips grew to confluency in LIF containing medium, they were observed under the AFM with an inverted optical microscopy underneath. AFM nanoindentation was performed on the center of mES cells. At each time point (0, 12, 18, 24 and 36 hrs) post RA addition, 50 cells were randomly selected and measured to obtain the force-displacement curves. In the AFM nanoindentation experiment, a cone-shaped AFM tip with a silicon nitride cantilever (Bruker AXS, Santa Barbara, CA) was used with the spring constant 0.06 nN/nm. The applied force was calculated by Hooke's law as 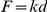, where 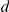 is the vertical deflection of the cantilever and 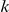 is the spring constant of the cantilever. According to Hertzian model, the relationship between indentation force and deformation defines (for a cone-shaped tip): 
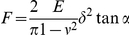
, where 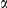 is the half angle of the cone-shaped AFM tip, 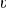 is the Poisson ratio, 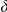 is the indentation depth and 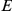 is the Young's modulus value. The half open angle of the tip in our case is 17.5° and we used 0.5 as Poisson ratio. By fitting the force-displacement curve with this model, Young's modulus value can be generated. The Young's moduli were then processed statistically to obtain the mean and standard deviation at each time point.

### Trypan blue exclusion assay

Sag^+/+^ or Sag^−/−^ mES cells were plated in 24-well gelatin-coated plate and incubated overnight at 37°C. Cells were treated next day with 1 µM RA for 24 or 36 hrs. Cells were detached with trypsin, stained with trypan blue dye, and counted microscopically. Ratio of dead blue cells out of total cells counted was plotted.

### DNA fragmentation analysis

The mES cells were seeded in 10-cm dishes at 2.5×10^6^ cells per dish and treated next day with DMSO (control) or RA (1 µM) for 36 hrs. The AML cells were seeded in 25-cm^2^ flasks at 0.5×10^6^ cell/mL (10 mL per flask) and treated for 24 hrs with DMSO (control), RA (1.5 µM), MLN4924 (0.1 µM for HL-60, 1.0 µM for KG-1), alone or in combination. Cells were harvested, pelleted, and lysed in 600 µl of lysis buffer (5 mM Tris-HCl, pH8.0, 20 mM EDTA, and 0.5% Triton X-100). DNA was isolated by phenol/chloroform extraction and ethanol precipitation and treated with RNAse and subjected to electrophoresis in a 1.8% agarose gel [14].

### Caspase activation assay

The activity of caspase3 was measured using a fluorogenic caspase assay with Ac-DEVA-AFC as the substrate. The results are expressed as fold activation, as compare to the control [14].

### TUNEL staining

Mouse embryonic stem cells were seeded in 12-well gelatin-coated plates, treated with DMSO vehicle control or 1 µM RA for 36 hrs. Cells were washed with cold PBS 3 times and fixed in 4% PFA-PBS for subsequent TUNEL staining using *in situ* Cell Death Detection Kit (Roche), according to manufacturer's instruction.

### Immunoblotting analysis

AML cells were harvested and lysed in a Triton X-100 lysis buffer (20 mmol/L Tris-HCl (pH 8.0), 150 mmol/L NaCl, 1% Triton X-100, 5 mmol/L EGTA, and 5 mmol/L EDTA) with freshly added protease inhibitor tablet (Roche, Indianapolis, IN) for 30 min on ice, followed by centrifugation for 20 min. Supernatants were measured for protein concentration using a Bio-Rad protein assay reagent (Bio-Rad, Hercules, CA) and subjected to immunoblotting analysis with the following antibodies: SAG monoclonal antibody (raised against the RING domain) [17], Caspase-3, CUL1, CDT1, β-Catenin, WEE1, IκBα, C-JUN (Santa Cruz Biotechnology, CA), β-Actin (Sigma, MO), p27 (BD PharMingen, CA), Phospho-IκBα, NOXA, PARP, BIM (Cell Signaling Technology, MA).

### FACS (Fluorescence-activated Cell Sorting)

Cells seeded at 0.5×10^6^ cell/mL in flasks (10 mL per 25-cm^2^ flask) were treated for 24 hrs (HL-60) or 36 hrs (KG-1) with DMSO (control), RA (1.5 µM), MLN4924 (0.1 µM for HL-60, 1.0 µM for KG-1), alone or in combination and analyzed by flow cytometry [17].

### Statistical analysis

Paired two-tailed Student's *t* test was used in comparisons between two groups to determine *p* value for statistical significance.
